# Military Surgery—Its Teaching in the Medical Schools

**Published:** 1862-09

**Authors:** 


### EDITORIAL DEPARTMENT.

### MILITARY SURGERY—ITS TEACHING IN THE MEDICAL SCHOOLS.

Present appearances indicate, so far as we are able to judge, that the army will hereafter afford a wide and permanent field for medical practice. The pressure of demand, and the excitement of raising volunteers, have in some instances operated upon medical men, and in their eagerness to serve their country, they have left homes to which they will desire to return when circumstances will permit, leaving their places to be supplied by others. They have been "mustered into service without drill," and constitute the "Militia" of the Army Medical Staff.

Young, able bodied and able minded men, between the ages of twenty-one and forty-five years, will be acceptable as military practitioners, provided always, they be sound and well qualified. The necessity of proper qualification has been made fully apparent by the experiences of the past, and the whole country are being aroused upon this subject. Thus far it will not be denied that inexperienced and incompetent men have found place, and been allowed to practice, neither diminishing the rate of mortality or adding to the respect and confidence which should repose in the army medical staff. While truth compels us to say this, yet we regard it as an exception to the general rule, and would be the last to depreciate the services or ability of that noble army of medical men, who have shown not only marked intelligence and capacity, but most heroic bravery, pursuing their mission under obstacles and amid dangers, which would have overwhelmed and crushed a less manly and resolute purpose.

If it has been true that joining the army indicated absence of, and often incapacity for, business at home; or, if in any instances, political influences have been allowed to take precedence in the appointment of military surgeons, we hope and believe that the time is near when qualification will be the only criterion, the standard so high as to make appointment in the United States Volunteer Army, a crown of honor. Military surgery as a distinct branch of medical knowledge, has not as yet been carefully studied in this country, and not until recently has the attention of the profession been directed to its magnitude and importance. While peace remained unbroken the implements of war were quite unused; so, while physicians were called to perform only civil service, the principles of military practice, or rather the principles of medicine and surgery as applicable to military practice, were quite neglected. Those who have accepted positions in the army, have grasped the idea at once of increasing their stock of ready knowledge, of obtaining fixed and definite ideas upon the various branches of military practice. Practical anatomy, or surgical anatomy, has been re-learned, surgical operations studied with a new interest, and everything connected with the more common ones, decided upon with definite purpose.

This independent knowledge, available at all times, without consultation or reference to books, is one of the necessities of military practice, and constitutes as broad line of distinction as can truthfully be drawn. We have often been asked to explain the differences in civil and military practice; to point out the particular qualifications required of the one, not equally important for the other. It may be we are unsuited to judge, since we have never "passed through the wars," and can hardly be expected to estimate accurately the duties and responsibilities of a business we have never followed.

In connection with the item of independent knowledge, already referred to, add to the qualifications of the well educated practitioner in civil life, thorough acquaintance with the hygiene of the military camp and hospital, and a full appreciation of its importance to the health and efficiency of the army, and we believe we have indicated the distinguishing characteristics which are claimed, and do actually exist. Military surgery is being learned and written in this country, and many facts and records of vital importance are rapidly collecting, which already greatly influence present practice. This is observable, not so much in the new modes of operation or treatment, as in hesitancy to adopt secondary operative interference, and improved sanitary regulations with better care and provision for the sick and wounded.

We have previously expressed the opinion, that fewer differences existed in civil, and military practice, than was generally supposed. Perhaps it will not be unfair or untrue to acknowledge that civil practitioners generally thought they knew a great deal more about military practice than they actually did. The results of secondary operations, as performed in our military hospitals, have no doubt disappointed the expectations of our best and most judicious surgeons. Our military surgeons were equally unable to predict or foresee the depressing agencies or influences which were to operate so unfavorably as to discourage as much as possible all judicious surgeons from making secondary operations, and in this were not a whit ahead of civil practitioners. As we have said, military surgery is being learned; and we hold ourselves open to its teachings, with the honest purpose of acknowledging our errors and correcting our opinions. If we have under-estimated the peculiarities of camp, field, or hospital practice, we have only erred in common with the profession generally, and are ready to speak frankly and freely upon all points of interest which the army experience is showing to exist.

Medical Colleges will provide for thorough instruction in this department, since the young men of the profession and medical students are eagerly looking to the army for the various positions which it offers to active, capable, and ambitious medical men. The impression is growing to be universal that it is almost a distinct science, if not in its general principles, at least in their application. Some institutions have already announced their Professorships of Military Surgery, and others will no doubt see the propriety and necessity of impressing its peculiarities and widely varying applications, by the oral teaching and illustration of a living Apostle,—

Nothing can fully satisfy the thirst for instruction in this department which does not pass by its name, and fully embody its principles.

Macleod, in his Notes on the Surgery of the Crimean War, says, "that Military Surgery does not differ from the surgery of civil life, is an assertion which is true in letter, but not in spirit. As a science, surgery is one and indivisible; but as an art, it varies according to the peculiar nature of the injuries with which it has to deal, and with the circumstances in which it falls to be exercised." He then speaks of the differences in camp practice, unlike in many respects, that which is ever presented to the practitioner in civil life, while not a few of the cases which are daily treated in domestic life rarely come under the charge of the military surgeon. The two classes of practitioners are said to be engaged in separate departments of the same profession, which, though uniting occasionally are yet distinct from each other. After pointing out other differences in the employment of the military surgeon, and noticing the influence of external circumstances, of extremes of climate, variations of food, work and shelter on the same men, as well as the effects of mental causes, as seen in the exultation of victory, and the prostration and dejection of defeat, he finally pays the following tribute: "If in war the surgeon sees much which is terrible, much which taxes his feelings of humanity, and his regret at the feebleness of his art, he has also the comforting conviction that nowhere is his beneficent mission so much felt, nowhere is the saving power of his profession so fully exercised; so true is it that chirurgery triumphs in armies and in sieges. 'Tis there that its empire is owned; 'tis there that its effects, and not words, express its eulogium."

